# Parasocial Engagement With Social Media Influencers and Mental Health Outcomes: Systematic Review and Meta-Analysis

**DOI:** 10.2196/96331

**Published:** 2026-08-20

**Authors:** Yixuan Li, Zihao Liu, Fangqing Liu

**Affiliations:** 1 School of Health in Social Science, University of Edinburgh, Edinburgh, Scotland, United Kingdom; 2 School of Business, University of Leicester, Leicester, United Kingdom; 3 Division of Psychology and Mental Health, School of Health Sciences, Faculty of Biology, Medicine and Health, University of Manchester, Booth Street East, Manchester, England, M13 9PL, United Kingdom, 44 07821867876

**Keywords:** meta-analysis, social media, mental health, parasocial relationships, parasocial interactions

## Abstract

**Background:**

Social media influencers occupy a pervasive role in billions of users’ daily digital lives, particularly among adolescents and young adults. Audiences develop parasocial engagement with these figures, including parasocial relationships (PSRs) and parasocial interactions (PSIs). Despite growing concern about their mental health implications, no prior meta-analysis has quantitatively synthesized this evidence.

**Objective:**

This systematic review and meta-analysis aimed to estimate the associations between influencer-directed parasocial engagement and mental health outcomes, examine prespecified moderators, and evaluate the quality of the existing evidence base.

**Methods:**

Seven databases (PsycINFO, Embase, MEDLINE, ERIC, PubMed, Web of Science, and Scopus) were searched from inception. Studies quantitatively assessing PSRs or PSIs with social media influencers and reporting mental health outcomes were eligible. A 3-level random-effects meta-analysis was conducted using Pearson *r*. Primary pooled estimates were calculated separately for positive or adaptive outcomes, and negative or maladaptive outcomes. Moderators examined included outcome domain, parasocial construct type, age group, gender, cultural region, and platform.

**Results:**

Seventeen studies (52 effect sizes) were included. Parasocial engagement was positively associated with both positive (*k*=28; *r*=0.38, 95% CI 0.18-0.55) and negative outcomes (*k*=24; *r*=0.24, 95% CI 0.08-0.38), with positive effects significantly stronger. Well-being showed the largest effects (*r*=0.42), followed by social media addiction (*r*=0.33). PSI demonstrated stronger associations than PSR (*r*=0.56 vs 0.22). Effects were largest among adolescents (*r*=0.44) and in Eastern samples (*r*=0.61 vs 0.29). No evidence of publication bias was detected (Egger *P*=.94; fail-safe N=18,643).

**Conclusions:**

Parasocial engagement functions as a psychological double-edged sword, reliably linked to both enhanced well-being and problematic engagement. Positive mental health outcomes, particularly those involving well-being, were generally stronger and more consistent than negative ones. These findings suggest that parasocial engagement should not be understood as uniformly beneficial or harmful; rather, its psychological meaning depends on the outcome domain, parasocial construct type, developmental stage, and cultural context. Future longitudinal, mechanism-based research with diverse samples is needed to clarify when parasocial engagement is most beneficial or harmful.

## Introduction

### Background

Over the past 2 decades, social media platforms have fundamentally reshaped the psychosocial environments in which people navigate daily life [1-3]. With more than 5 billion active users worldwide as of 2024 [4], platforms such as Instagram (Meta Platforms, Inc), TikTok (ByteDance Ltd), YouTube (Google LLC), and X (formerly Twitter; X Corp) constitute not merely communication technologies but pervasive digital environments that mediate identity formation, social comparison, affect regulation, and interpersonal connection [5-7]. Within these environments, a distinctive and increasingly prominent class of media figures has emerged: social media influencers.

Unlike traditional celebrities whose visibility is mediated through broadcast formats and institutional gatekeepers, influencers establish continuous, intimate, and algorithmically amplified presences in the everyday digital lives of their audiences, disclosing aspects of their personal circumstances, relationships, and emotional experiences with a frequency and granularity that has no clear precedent in the history of mass media [8,9]. Estimates suggest that the global influencer marketing industry exceeded US $21 billion in 2023 [10]; yet, the psychological consequences of audiences’ sustained engagement with these figures remain poorly characterized. Given the scale and intimacy of influencer exposure, particularly among adolescents and young adults, for whom influencer content constitutes a primary source of social information [11], lifestyle reference [12], and emotional stimulation [13], the mental health implications of the relational bonds that audiences form with such figures are a pressing public health concern [14].

### Theoretical Framework

A theoretically coherent framework for understanding the audience-influencer bond is provided by the construct of parasocial relationships (PSRs) [15]. First formalized by Horton and Wohl [16], PSRs are defined as stable, affectively charged, and subjectively intimate connections that audience members develop toward media personae, which are connections that mimic the phenomenology of genuine social relationships while remaining structurally 1-sided and unreciprocated [17]. Subsequent research has distinguished PSRs from the more immediate and episodic phenomenon of parasocial interaction (PSI), situating PSRs as dispositional relational schemata that persist across exposure episodes and influence how audiences attend to, process, and affectively respond to media figures [18,19]. Prior conceptual work has therefore used “parasocial engagement” as a collective term for related parasocial phenomena, including both PSI and PSR [20]. Consistent with this usage, the present review uses parasocial engagement as an umbrella term for PSRs, PSIs, and multidimensional parasocial engagement.

Although parasocial engagement was initially theorized with reference to television personalities and fictional characters, the distinctive affordances of social media platforms have substantially altered the conditions under which such bonds form and intensify [17,21]. Influencers’ practices of unfiltered self-disclosure, behind-the-scenes access, direct comment section interaction, and live streaming create a sustained perceptual ecology of availability and reciprocity that substantially exceeds what broadcast media could generate [22]. Algorithmic recommendation systems further ensure high-frequency and contextually personalized exposure, consolidating the subjective sense of a shared, evolving relational history [23]. The cumulative result is that parasocial engagement formed with social media influencers may be qualitatively more intense, more cognitively pervasive, and more emotionally consequential than those documented in earlier media contexts, which is a possibility with direct implications for mental health [17,24,25]. Critically, influencer-directed parasocial engagement is conceptually distinct from more distal predictors such as general social media use duration or passive platform browsing as it captures the degree of relational investment in a specific media figure and thus represents a more proximal and mechanistically interpretable exposure variable [19].

From a theoretical standpoint, the relationship between influencer-directed parasocial engagement and mental health outcomes is unlikely to be uniformly valenced; rather, converging theoretical traditions support the plausibility of both risk and protective pathways [26]. On the risk side, social comparison theory [27] indicates that repeated exposure to the idealized self-presentations characteristic of many influencers, particularly in domains of physical appearance and material success, may activate upward comparisons that generate body dissatisfaction, perceived relative deprivation, and diminished self-worth [6,28,29]. The incorporation of parasocial engagement into this dynamic may amplify these effects: insofar as audiences construe an influencer as a quasi-intimate reference point rather than a distal media figure, invidious comparisons may be experienced with heightened personal relevance and emotional intensity [19,30].

Self-discrepancy theory [31] further predicts that chronic exposure to influencer-modeled ideal-self standards will engender negative affect states, such as anxiety and depression, when individuals perceive a significant gap between their actual and ideal selves [32]. Beyond comparison-based mechanisms, strong parasocial engagement may foster forms of emotional dependency that render individuals vulnerable to relational frustration: however, if parasocial engagement replaces rather than supplements offline social connection—a compensatory pattern anticipated by uses and gratifications theory—it may ultimately intensify loneliness and leave belongingness needs unmet [33]. Evidence from body image research is broadly consistent with these theorized risk pathways, with systematic reviews documenting reliable associations between social media use and body dissatisfaction [34-37], and converging evidence linking heavy social media engagement to elevated depressive symptoms, anxiety, and psychological distress, particularly among women and adolescents [38,39].

Protective pathways are nonetheless equally theoretically grounded. Parasocial engagement with influencers who engage in authentic self-disclosure, mental health advocacy, or normalization of personal struggle may provide companionship, perceived social support, and validated experience, which are resources that could attenuate loneliness, reduce help-seeking stigma, and support adaptive coping among individuals with limited offline social networks [17,33,40,41]. From a social compensation perspective, individuals who experience deficits in face-to-face social connection, including those with social anxiety, minority status, or restricted community access, may be particularly motivated to form and sustain parasocial bonds as a means of fulfilling unmet needs for belonging and affiliation [42]. Rather than representing maladaptive substitution, such compensatory engagement may confer genuine psychological benefit when offline relational resources are structurally constrained. Complementing this account, self-determination theory [43] offers a motivational framework through which parasocial engagement may satisfy 3 basic psychological needs, such as autonomy, competence, and relatedness, with the need for relatedness being particularly relevant in the context of parasocial engagement with influencers. When PSIs are experienced as warm, nonjudgmental, and affirming, they may partially fulfill relatedness needs in ways that support intrinsic motivation for self-care and help-seeking behavior [44]. For marginalized groups or individuals in formative stages of identity development, parasocial bonds with influencers who model inclusive or affirmative identities may further function as developmental scaffolding, supporting self-acceptance and group belonging in ways that carry salutogenic implications [45]. That both pathways are plausible and empirically attested underscores the need to evaluate influencer-parasocial engagement across a range of mental health outcomes rather than presupposing a single direction of association [46].

These theoretical perspectives suggest that parasocial engagement with social media influencers may be associated with different mental health–related outcomes, depending on different pathways [20]. Some pathways are primarily affective and relational, such as perceived companionship, emotional support, and relatedness, and are therefore most relevant to well-being outcomes, including psychological well-being, life satisfaction, and quality of life [12,47]. Other pathways concern self- and social functioning, such as self-evaluation, perceived competence, and prosocial mental health–related responses, which are reflected in outcomes such as self-esteem, self-efficacy, and willingness to support mental health resources [48,49]. At the same time, parasocial engagement may also be linked to behavioral patterns of repeated checking, continued exposure, and emotional investment in influencer content, making problematic or addictive social media use a theoretically relevant outcome domain [50,51]. Finally, comparison-based and discrepancy-based pathways may be linked to psychological distress and maladaptive cognitive-affective responses, such as envy, social comparison, depressive symptoms, anxiety, and maladaptive schemas [52,53]. Therefore, the present review conceptualizes the included outcomes as 4 domains: Well-Being, Positive Functioning, Problematic Social Media Use, and Psychological Distress.

Despite the theoretical significance of this question, the empirical literature examining associations between influencer-directed parasocial engagement and mental health outcomes is characterized by substantial heterogeneity, disciplinary fragmentation, and methodological variability. Studies have variously reported negative associations between parasocial engagement strength and outcomes including body satisfaction, self-esteem, and depression; positive associations between parasocial engagement and subjective well-being, social connectedness, or reduced loneliness; and null or context-dependent relationships [54,55]. This pattern of inconsistency is likely attributable to several intersecting sources of variance. Without systematic synthesis across this dispersed and heterogeneous corpus, the true direction, magnitude, and boundary conditions of the association between influencer-directed parasocial engagement and different mental health outcomes remain unclear [21,33,48].

### Potential Moderators

The association between influencer-directed parasocial engagement and mental health outcomes is unlikely to be uniform across studies; rather, substantive and methodological characteristics may moderate its magnitude and direction. Given the conceptual heterogeneity of both parasocial constructs and outcome domains, moderator analyses were used to examine whether associations differed across theoretically meaningful study characteristics. In particular, different outcome domains were examined to avoid treating diverse mental health-related outcomes as indicators of a single homogeneous construct.

#### Outcome Domain

The type of mental health–related outcome may shape the observed association between parasocial engagement and psychological functioning. Parasocial engagement may differentially relate to indicators of positive versus negative outcomes, insofar as the mechanisms linking parasocial engagement to benefit versus harm are theoretically distinct [17,33]. Examining outcome domain as a moderator is therefore essential to characterizing the boundary conditions of the overall effect. In the present review, outcomes were examined first by broad positive versus negative outcomes and then by 4 specific domains: Well-Being, Positive Functioning, Problematic Social Media Use, and Psychological Distress.

#### Type of Parasocial Construct

Although the present review uses parasocial engagement as an umbrella term [20], PSRs and PSIs represent conceptually distinguishable constructs, with the former referring to stable relational schemata and the latter to in-the-moment experiential engagement during media exposure [18,55]. This combination should not be interpreted as specific to either PSR or PSI alone. Because these constructs may operate through different affective and cognitive mechanisms, parasocial construct type was examined as a moderator and construct-specific subgroup estimates were reported where possible.

#### Age Group

The developmental context of media consumption may moderate the influence of parasocial engagement on mental health. Adolescents, who are at a formative stage of identity development and more susceptible to social comparison pressures, may be particularly sensitive to the relational dynamics of influencer engagement [5]. Younger audiences may therefore show stronger associations between influencer-directed parasocial engagement and both risk and protective mental health outcomes relative to adults.

#### Gender

Gender may also shape the association between influencer-directed parasocial engagement and mental health. Women and girls are disproportionately exposed to appearance-focused influencer content and show greater vulnerability to upward social comparisons in digital contexts [6,39], which may strengthen associations between influencer-directed parasocial engagement and mental health outcomes in female samples.

#### Cultural Region

Cultural context may moderate how audiences invest in and are psychologically affected by parasocial bonds. Differences in collectivist versus individualist self-construals may influence both the intensity of parasocial engagement formed with influencers and the behavioral and emotional responses that follow [19]. Eastern and Western cultural contexts may therefore yield divergent effect sizes.

#### Social Media Platform

The affordances of specific platforms, including algorithmic recommendation intensity, content format, and interaction norms, may shape the depth of parasocial engagement and its downstream psychological consequences [22,23]. Differences across platforms such as Instagram, TikTok, and YouTube may therefore contribute to between-study heterogeneity.

### The Present Study

Prior narrative and systematic reviews have made important contributions to adjacent literatures by documenting the effects of broadly defined social media use on body image [56], well-being [57], and disordered eating behaviors [58]; yet, none has specifically and comprehensively synthesized the evidence on PSRs with social media influencers as an associative factor of mental health outcomes. The absence of such evidence represents a significant gap, as claims that this form of engagement offers meaningful psychological benefits are not yet supported by robust empirical evidence.

Therefore, the present systematic review and meta-analysis addresses these gaps by providing the first comprehensive quantitative synthesis of empirical research on the associations between parasocial engagement with social media influencers and mental health outcomes. Three research objectives guide this work:

- Research objective 1: To estimate the pooled effect size of the association between influencer-directed parasocial engagement and both positive and negative mental health outcomes across the available empirical literature.
- Research objective 2: To examine the extent and sources of between-study heterogeneity through prespecified moderator analyses, including mental health outcome domain, type of parasocial engagement (PSR vs PSI), social media platform, participant age, and gender.
- Research objective 3: To critically evaluate the quality of the current evidence base and identify priority areas for future research.

## Methods

### Protocol and Registration

This systematic review and meta-analysis was conducted and reported in accordance with the PRISMA (Preferred Reporting Items for Systematic Reviews and Meta-Analyses) guidelines [59] (Checklist 1). The review protocol was prospectively registered on PROSPERO (registration number CRD420261291637) prior to the commencement of data collection.

### Eligibility Criteria

Eligibility was operationalized using the Population, Exposure, Comparator, and Outcomes framework (Table 1). Studies were included if they met all the following criteria and excluded if they met any of the exclusion criteria specified in Table 1.

**Table 1.** Population, Exposure, Comparator, and Outcomes framework for eligibility criteria.

Component	Eligibility criteria
Population (P)	Social media users of any age, including adolescents, young adults, and adults. No restrictions were placed on country of origin, cultural context, or sampling method.
Exposure or intervention (E)	Quantitative assessment of parasocial engagement with social media influencers. Influencers were defined as individuals who create and disseminate content on social media platforms and maintain an audience through sustained online presence and perceived influence. No restrictions were placed on platform (eg, Instagram, TikTok, and YouTube) or influencer genre.
Comparison (C)	Not required. Eligible studies were not required to include a comparison group, as the review focused on the association between influencer-directed parasocial engagement and mental health outcomes across observational and experimental designs.
Outcomes (O)	At least 1 quantitatively measured mental health outcome. Primary outcomes included loneliness, depression, and anxiety, assessed using validated or widely used self-report measures. Secondary outcomes included broader indicators of psychological well-being, such as self-esteem, life satisfaction, subjective well-being, body satisfaction, and psychological distress.
Study design (S)	Empirical quantitative studies using cross-sectional, longitudinal, or experimental designs. Studies had to report sufficient statistical information to permit effect size calculation or extraction of equivalent data (eg, correlations, regression coefficients, means, and standard deviations, or other compatible statistics).
Language	English-language studies only.
Exclusion criteria	Studies were excluded if they (1) focused exclusively on parasocial relationships with traditional celebrities without a social media influencer context; (2) measured only general social media use or platform engagement without a parasocial engagement construct; (3) used qualitative, case study, editorial, theoretical, or narrative review designs; (4) did not provide sufficient quantitative data to compute or estimate an effect size; or (5) were conference abstracts, book chapters, or other non–peer-reviewed formats without a dissertation or preprint equivalent.

### Information Sources and Search Strategy

A comprehensive electronic search was conducted across 7 bibliographic databases: PsycINFO, Embase, MEDLINE, ERIC, PubMed, Web of Science, and Scopus. Searches were conducted from database inception to the date of the final search. To supplement electronic database searching and mitigate retrieval bias, the first 200 results of a Google Scholar search (sorted by relevance) were manually screened. Reference lists of all retained full-text papers and relevant systematic reviews in the adjacent literature were hand-searched. Authors of potentially eligible studies were contacted to request missing statistical data where necessary. The search strategy was developed iteratively in consultation with the research team and was structured around three conceptual domains: (1) the parasocial construct, (2) social media influencers, and (3) mental health outcomes. The full database-specific search strings, including controlled vocabulary terms (eg, MeSH headings), are reported in Multimedia Appendix 1.

### Study Selection

All records retrieved from database searches were imported into Rayyan systematic review management software (Qatar Computing Research Institute, Hamad Bin Khalifa University), where automatic deduplication was performed. Remaining duplicates were identified and removed manually before screening. Title and abstract screening and full-text review were conducted independently by 2 reviewers (FQL and YXL). During title and abstract screening, records were assessed for prima facie eligibility using a standardized screening form operationalizing the prespecified Population, Exposure, Comparator, and Outcomes criteria. Full texts of all records passing title and abstract screening were retrieved and independently reviewed against the complete eligibility criteria. Disagreements at both screening stages were resolved through structured discussion. Interrater reliability at both stages was quantified using Cohen κ, with κ≥0.80 designated as the threshold for acceptable agreement [60].

### Data Extraction

Data were extracted using a standardized form piloted on 5 studies; discrepancies were resolved by discussion or third-party adjudication. Multiple effect sizes from the same study were treated as dependent and handled as described in the Data Synthesis section. Missing data were sought from corresponding authors via up to 2 email requests over 4 weeks. Extracted information included (1) *study characteristics* (authors, year, country, design, and sampling method), (2) *sample characteristics* (sample size, mean age, age range, gender distribution, and clinical vs community status), (3) *exposure characteristics* (PSR vs PSI construct, measure, item count, reliability, platform, and influencer type), (4) *outcome characteristics* (mental health outcome type, measure, item count, and reliability), and (5) *statistical information* (correlations, standardized and unstandardized regression coefficients, means, standard deviations, and any statistics necessary for effect size computation).

### Risk-of-Bias Assessment

Methodological quality was assessed independently by 2 reviewers, with disagreements resolved through discussion. Study quality tools were selected according to study design. The Joanna Briggs Institute (JBI) Critical Appraisal Checklist for Analytical Cross-Sectional Studies [61] was applied for cross-sectional studies. This instrument evaluates criteria including sample representativeness, validity of exposure and outcome measurement, appropriate statistical methods, and adequate control of confounding. For each study, an overall methodological quality score was calculated as the proportion of criteria rated as adequately met.

### Data Synthesis and Statistical Analysis

#### Narrative Synthesis

Before conducting the meta-analysis, we first conducted a structured narrative synthesis to map the range of mental health outcomes assessed across the included studies and to organize them by conceptual meaning. This synthesis followed the Synthesis Without Meta-Analysis reporting guidelines [62]. Outcomes were first grouped by broad valence, distinguishing positive from negative outcomes, and were then organized by more specific outcome domains. These domains included well-being outcomes, positive functioning and prosocial response outcomes, problematic or addictive social media use outcomes, and negative affective or maladaptive social-cognitive outcomes. Following this narrative synthesis, 3-level meta-analyses were conducted for outcome categories with sufficient available effect sizes. Statistical pooling was conducted separately for positive and negative outcomes, and more specific domain-level analyses were conducted where possible. For outcome categories where fewer than 3 studies were available, or where statistical pooling was considered inappropriate due to excessive outcome heterogeneity, findings were retained in the narrative synthesis rather than pooled quantitatively.

#### Effect Size Metric

Pearson product-moment correlation coefficient (*r*) was adopted as the common effect size metric, consistent with conventions in the social and health psychology literature. Where studies reported effect sizes in other metrics (eg, standardized regression coefficients β, Cohen *d*, and odds ratios), these were converted to *r* using established formulae [63]. All *r* values were transformed to Fisher *z* prior to analysis to stabilize variance and normalize the sampling distribution; pooled estimates were backtransformed to *r* for reporting. Effect sizes were interpreted using the conventional benchmarks of *r*=0.10 (small), *r*=0.30 (medium), and *r*=0.50 (large).

#### Meta-Analytic Model

Because multiple effect sizes were extracted from several studies (eg, different outcomes or dimensions measured within the same sample), effect sizes were not statistically independent. A 3-level random-effects meta-analysis was therefore adopted as the primary analytic model to account for the nested structure of the data, with sampling variance at level 1, effect sizes nested within studies at level 2, and between-study variance estimated at level 3. This approach allows for the simultaneous estimation of within-study and between-study variance components and is recommended when multiple effect sizes are obtained from the same study [64].

Primary pooled estimates were calculated separately for positive and negative outcomes because positive correlations have different substantive meanings depending on outcome valence. Positive correlations with adaptive outcomes, such as well-being or life satisfaction, indicate more favorable psychosocial functioning, whereas positive correlations with maladaptive outcomes, such as problematic social media use, addiction, or psychological distress, indicate greater difficulties. To provide a descriptive summary of the broader evidence base and to support moderator analyses of heterogeneity, we also estimated an omnibus model including all eligible effect sizes. This omnibus estimate was treated as a secondary descriptive analysis and was not interpreted as a single beneficial or harmful mental health effect.

Model fit was evaluated by comparing the 3-level model against a conventional 2-level model using the Akaike Information Criterion (AIC) and likelihood ratio tests. All analyses were conducted in R (version 4.5.1; R Core Team) [65] using the metafor package [66], with the restricted maximum likelihood estimator used for between-study variance components (τ²). Where the number of studies per analysis was insufficient for robust estimation (*k*<5), results were interpreted with appropriate caution and sensitivity analyses under alternative variance estimators (eg, Hedges and Paule-Mandel) were conducted.

#### Assessment of Heterogeneity

Between-study heterogeneity was quantified using the *Q* statistic (significance threshold set at *P*<.10 to reflect the reduced power of the *Q* test with small *k*), the *I*² statistic (the proportion of total variability attributable to true between-study heterogeneity), and the τ² estimate (the estimated variance of the true effect size distribution). Following Higgins et al [67], *I*² values of approximately 25%, 50%, and 75% were interpreted as indicative of low, moderate, and high heterogeneity, respectively. Variance was decomposed into between-study and within-study components. Prediction intervals (95% prediction intervals) were additionally computed to convey the range within which the true effect size is expected to fall in 95% of comparable future studies, providing a more practically interpretable index of effect variability than *I*² alone.

#### Moderator Analyses

To examine potential sources of between-study heterogeneity, a series of prespecified moderator analyses were conducted using mixed-effects meta-regression models within the 3-level framework. Categorical moderators were examined via subgroup analyses; continuous moderators were examined via weighted meta-regression with restricted maximum likelihood estimation. Moderator analyses were conducted only where a minimum of *k*=3 effects was available per subgroup to avoid unreliable estimates in small samples. Pseudo-*R*² values were calculated for continuous moderators to index the proportion of between-study variance accounted for by each predictor.

Given that the moderator analyses were prespecified and theoretically supported, each moderator was treated as addressing a distinct conceptual source of heterogeneity rather than as part of a single family of interchangeable post hoc comparisons. Therefore, a formal Bonferroni correction was not applied. Moderator findings were evaluated using uncorrected *P* values, with *P*<.05 considered statistically significant, alongside 95% CIs, where intervals not including zero were interpreted as providing evidence of an association.

The following moderators were prespecified in the PROSPERO protocol: (1) outcome domain (well-being, positive functioning, social media addiction, and psychological distress), (2) type of parasocial construct assessed (PSR vs PSI), (3) age group (adolescents [aged <18 years] vs young adults [aged 18‐25 years] vs adults [aged >25 years]), (4) gender composition of the sample (proportion female, treated as a continuous moderator), (5) social media platform studied (Instagram, TikTok, YouTube, multiplatform, or unspecified), and (6) study location (Western vs Eastern countries).

#### Assessment of Publication Bias and Small-Study Effects

The potential for publication bias and small-study effects was evaluated using a convergent multimethod approach, applied to any primary meta-analytic estimate based on *k*≥10 studies. Funnel plot asymmetry was visually inspected and formally tested using Egger regression test (*P*<.10). The trim-and-fill procedure was applied to estimate the number of potentially suppressed studies and to compute bias-adjusted pooled estimates, and the limit meta-analysis method was applied as a more robust alternative under conditions of heterogeneity. Selection model approaches were used as a further sensitivity check, testing whether results were robust under models that weight studies by their likelihood of publication as a function of their *P* value.

#### Sensitivity Analyses

A series of sensitivity analyses were conducted to assess the robustness of primary findings. Leave-one-out analyses were performed by sequentially removing each study and recalculating the pooled estimate to identify disproportionately influential studies. Analyses were additionally repeated restricting to studies rated as high methodological quality (JBI or Risk of Bias 2 score ≥70%), to studies using validated published PSR or PSI measures (as opposed to researcher-developed items), and to peer-reviewed journal papers (excluding gray literature). To examine whether results were influenced by converted effect sizes, analyses were rerun using only reported (unconverted) correlation coefficients, and effect size source was tested as a categorical moderator. Divergence between primary and sensitivity analyses is discussed in the context of effect size interpretation.

## Results

### Overview

**Figure 1.**
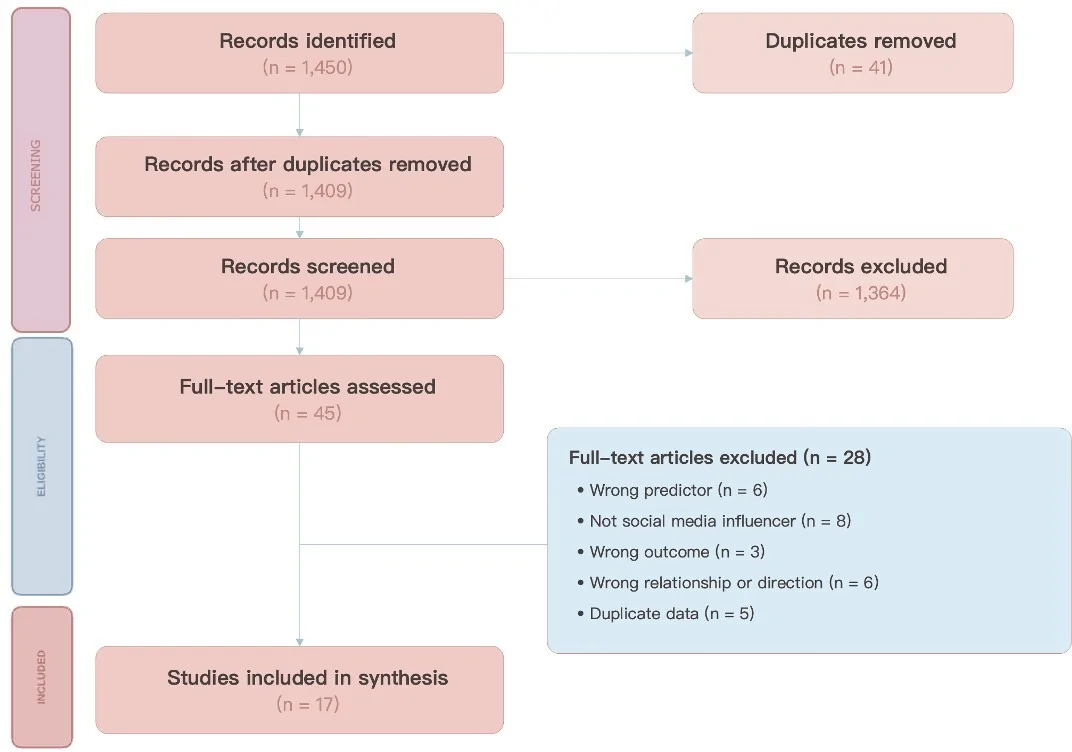
The PRISMA (Preferred Reporting Items for Systematic Reviews and Meta-Analyses) flowchart for study inclusion [59].

Initially, 1450 literature records were imported and 41 duplicates were removed. The remaining 1409 studies were screened by title and abstract, leading to the exclusion of 1364 studies. Of the 45 full-text papers assessed for eligibility, 28 were excluded. Ultimately, 17 studies were included in the review (Figure 1).

### Study Characteristics

Detailed characteristics are presented in Table 2. As shown in Multimedia Appendix 2, descriptive statistics are presented across several visualizations, including publication trends, study location, study design, parasocial construct type, social media platform, participant age group, mental health outcome type, and sample size distribution. All studies were published in English between 2018 and 2025, peaking in 2024‐2025, consistent with growing interest in influencer-based parasocial engagement. Most used cross-sectional surveys (n=14), with 1 experimental study and 2 mixed methods studies. Sample sizes ranged from 124 to 932. Participants were mainly adolescents, university students, or young adults; mean age ranged from about 12-33 years, and female representation ranged from 28% to 100%. Studies were conducted across the United States (n=6), China (n=3), France (n=2), and South Korea, Spain, Sweden, Poland, India, and North America (1 each). Most studies examined PSR (n=9) or PSI (n=7); 2 assessed broader multidimensional parasocial constructs. Measures were typically adapted from established instruments, such as the 10-item version of the PSI Scale [68,69], the Parasocial Attachment Scale [70], and the Multidimensional Measure of Parasocial Relationships [48]. Influencers included mixed-platform or virtual influencers (n=9), YouTubers (n=4), Instagram influencers (n=2), and TikTok creators (n=1).

**Table 2.** Study characteristics.

Study ID	Study design	Study location	Population characteristics				Parasocial characteristics		Influencer or platform characteristics		Mental health outcomes characteristics		Analytic information	
			Sample size, n	Mean age (years)	Age (years) or grade range (years)	Percentage of females	Parasocial construct (PSR^a^, PSI^b^, or other)	Parasocial measure or scale	Influencer type (YouTuber or TikTok creator or Instagram influencer or Streamer or Virtual influencer or Mixed)	Social media platform (YouTube or TikTok or Instagram or Mixed or Other)	Outcome type	Outcome measure or scale	Statistical model	Covariates or control variables
Bartosiak et al (2025) [12]	Cross-sectional study	United States	863	33	18- 40	42	PSI	12 items adapted from Brown and Bocarnea [71], modified to use the term influencer	Virtual influencer	Mixed	Social well-being, psychological well-being, and financial well-being	Six-item social well-being scale, 12-item psychological well-being scale, and 10-item CFPB^c^ Financial Well-Being Scale	Structural equation modeling	Age, gender, ethnicity, marital status, education, employment status, and household income
de Bérail et al (2019) [50]	Cross-sectional study	France	932	21.3	18+	72.90	PSR	An adapted version of the 10-item version of the Parasocial Interaction Scale	YouTuber	YouTube	YouTube addiction	Internet Addiction Test	Multiple regression analyses and structural equation modeling with bootstrap procedure	Age, sex, nationality, time spent last week watching YouTube videos, and a YouTube activity index
de Bérail and Bungener (2022) [51]	Cross-sectional study	France	370	19.62	18-29	N/A^d^	PSR	10-item PSI Scale adapted to YouTube, then reduced to 9 items and modeled as 3 dimensions: desire to engage in parasocial processes, feeling of intimacy, and feeling of attraction	YouTuber	YouTube	YouTube addiction	20-item YouTube-adapted Internet Addiction Test	Structural equation modeling; mediation model	Age, sex, social anxiety, loneliness, anxiety attachment, avoidance attachment, factual self-disclosure, evaluative self-disclosure, level of fictional content, level of physical presence, average length of videos, and average rhythm of publication
Chen et al (2024) [52]	Cross-sectional study	China	597	N/A	N/A	53.30	PSI	Parasocial interaction scale	Virtual influencer	YouTube	Online social well-being	Online social well-being scale	Structural equation modeling	Loneliness, low self-esteem, empathy, expertise, homophily, and social attractiveness
Chen et al (2025) [72]	Cross-sectional study	China	554	N/A	N/A	72	PSI	Parasocial interaction with entertainment-oriented influencers	YouTuber	YouTube	Hedonic well-being	Hedonic well-being scale	Structural equation modeling	Gender, age, average time spent per viewing session, and viewing frequency as moderator
Farivar et al (2022) [73]	Mixed methods study; Delphi study plus cross-sectional survey	North America	500	N/A	18+	53	PSR	Parasocial relationship with influencer	Instagram influencer	Instagram	Problematic engagement	Problematic engagement with influencers	Structural equation modeling	Gender, age, daily time spent on Instagram, and length of using Instagram
Garcia et al, (2022) [48]	Cross-sectional pilot study	Sweden	259	25.3	N/A	79.90	Other; affective, behavior, cognitive, and decisional dimension of parasocial relationships	Multidimensional Measure of Parasocial Relationships, 18 items, 4-point Likert scale	Mixed social media figures	Mixed	Social comparison; self-esteem	Iowa-Netherlands Comparison Orientation Measure, and the Rosenberg Self-Esteem Scale	Exploratory factor analysis, confirmatory factor analysis, and structural equation modeling	N/A
Hoffner and Cohen (2018) [49]	Cross-sectional study	United States	350	37.85	20- 82	60.57	PSR	Parasocial relationship with Robin Williams prior to his death	Celebrities	Mixed	Depression stereotypes, social distance, support for public mental health resources, willingness to seek treatment, shared mental health information, and offered mental health support	Social distance measure, support for public mental health resources, willingness to seek treatment, shared mental health information, and offered mental health support	Hierarchical regression analyses	Gender, age, education, political orientation, and experience with depression; overall media exposure and types of media exposure were also entered in later blocks
Jin (2018) [74]	Randomized experiment, 2×2 between-subjects design	South Korea	141	33.3	18+	100	PSI	PSI measured with 4 items	Instagram foodie	Instagram	Social media envy	Social media envy with 6 items; benign and malicious envy with 10-item envy scale	Two-way ANOVA; mediation analysis	Self-esteem, BMI, perfectionism, anorexia, and bulimia nervosa
Kim and Kim (2020) [75]	Cross-sectional survey	United States	642	N/A	20+	54	PSI	To measure all constructs for the current research, multiple items were adapted and revised from previous studies	Celebrities	Mixed	Quality of life; well-being	Quality-of-life and well-being scales	Structural equation modeling	N/A
Kim and Kim (2026) [76]	Cross-sectional study	United States	587	N/A	20+	52.50	PSI	Perceived friendship with virtual influencers	Virtual influencer	Mixed	Psychological well-being, social media engagement, and purchase intention	Psychological well-being, social media engagement, and purchase intention scales	Structural equation modeling	Age, gender, and social media usage (eg, duration of time spent on social media and frequency of interactions with VIs)^e^
Lee et al (2021) [53]	Cross-sectional study	United States	234	33.56	19+	32.60	PSI and PSR	We used a repurposed measure for parasocial relationship in the scenario of streaming viewing adapted from a 15-item 5-point scale on television by Rubin et al (1985) [69]. The operationalization of parasocial relationships was the viewers’ perceived friendship or relationship with the streamer.	Streamer	Mixed, Twitch or YouTube	Depression risk susceptibility, risk severity, and self-efficacy to seek help	Perceived prevalence was assessed as an outcome variable. Risk susceptibility and risk severity were measured with subscales from the Extended Parallel Process Model. Self-efficacy toward dealing with depression was measured using 3 items	Hierarchical regression	Frequency of watching the streamer, empathetic concern, personal distress, and depression
Martín-Cárdaba et al (2024) [77]	Correlational cross-sectional study	Spain	800	12.33	8-16	50	PSR	Parasocial relationship with the influencer	Mixed	Mixed	Psychological discomfort, problematic usage, and imitation of dangerous behaviors	Negative consequence measures for psychological discomfort, problematic usage, and dangerous behaviors	Moderated double mediation model using PROCESS Model 85, plus moderation analysis using PROCESS Model 3	Age and sex reported in the bivariate correlation table; ownership and active mediation were modeled as focal predictors or moderators
Ravi and Patki (2025) [78]	Mixed methods study, quantitative cross-sectional survey plus qualitative semistructured interviews	India	124 in the quantitative stage; 15 in the qualitative stage	20.6	18-24	68.50	PSR	Multiple Parasocial Relationships Scale	Celebrities	Mixed	Psychological well-being; perceived social support (qualitative)	Shortened 18-item version of the Psychological Well-Being Scale	Quantitative survey analysis plus thematic analysis of interviews	N/A
Wang and Shang (2024) [79]	Cross-sectional study	China	379	19.8	15-25	56.70	PSR	Six items adapted from the Parasocial Attachment Scale	TikTok creator	TikTok	Social media addiction and life satisfaction; TikTok fatigue	Life satisfaction and fatigue scales; exact scale names not fully reported in retrieved text	Structural equation modeling	Demographic items were collected; specific covariates not reported in retrieved text
Witkowska et al (2025) [80]	Cross-sectional validation study with 4-week test-retest subsample	Poland	371	23.22	18-48	68.70	Other; affective, behavior, cognitive, and decisional dimension of parasocial relationships	MMPR^f^, 18 items, 4-point Likert scale	Mixed	Mixed	Emotional experiences, maladaptive schemas	SPANE^g^ positive emotions, negative emotions, affective balance, and selected early maladaptive schemas	Confirmatory factor analysis, reliability analysis, test-retest correlations, and convergent validity correlations	N/A
Wolff and Shen (2024) [81]	Cross-sectional survey	United States	396	N/A	18+	28	PSR	Thirteen items measured participants’ PSR with their favorite streamer	Streamer	Twitch	Psychological well-being	Brief Inventory of Thriving	Structural equation modeling with bias-corrected bootstrapped confidence intervals	Time spent watching favorite streamer, perceived offline social support, education, health, and sex

a PSR: parasocial relationship.

b PSI: parasocial interaction.

c CFPB: Consumer Financial Protection Bureau.

d N/A: not applicable.

e VIs: virtual influencers.

f MMPR: Multidimensional Measure of Parasocial Relationships.

g SPANE: Scale of Positive and Negative Experience.

### Narrative Synthesis of Positive and Negative Mental Health Outcomes

Before conducting the meta-analysis, we first conducted a structured narrative synthesis to map the range of mental health–related and psychosocial outcomes assessed across the included studies. Given the conceptual breadth of the included outcomes, outcomes were first classified by positive or negative outcomes. They were then further organized into four conceptually meaningful domains: (1) Well-Being, (2) Positive Functioning, (3) Problematic Social Media Use, and (4) Psychological Distress.

#### Positive Outcomes

##### Well-Being

The first positive domain included indicators of well-being, such as psychological well-being [12,75,76,78,81], quality of life [75], social well-being [12,52], hedonic well-being [72], life satisfaction [75,79,81], and positive emotions or affective balance [80]. Across these studies, parasocial engagement with social media influencers was generally positively associated with well-being–related outcomes. However, the strength and consistency of associations varied, with some outcomes showing small or nonsignificant associations, including overall well-being [78] and psychological well-being [81].

##### Positive Functioning

The second positive domain included outcomes reflecting positive self-evaluation, perceived competence, and prosocial mental health–related responses. These outcomes included high self-esteem [48], willingness to support public mental health resources [49], and self-efficacy in dealing with depression [53]. Findings in this domain were less consistent than those for well-being outcomes. Some studies reported positive associations, whereas others found small or nonsignificant associations, including support for public mental health resources [49] and self-efficacy in dealing with depression [53].

### Negative Outcomes

#### Problematic Social Media Use

The first negative domain included outcomes related to problematic or addictive engagement with social media platforms. These included YouTube addiction [50,51], problematic engagement or usage [73,77], and TikTok fatigue or social media addiction [79]. Across these studies, stronger parasocial engagement was generally associated with higher levels of problematic or addictive social media use, although the magnitude and consistency of associations varied across platforms and outcome measures. Some associations, such as those involving TikTok fatigue [79], were nonsignificant.

#### Psychological Distress

The second negative domain included psychological distress and negative cognitive-affective responses, such as depression-related perceptions [49,53], psychological discomfort and risky behaviors [77], social media envy [74], social comparison [48], and negative emotions or maladaptive schemas [80]. In most cases, stronger parasocial engagement was associated with higher levels of emotional distress or maladaptive cognitions. However, findings were not uniform. Some studies reported lower levels of certain negative outcomes, such as social distance [49] and perceived susceptibility to depression [53], whereas several associations were nonsignificant, including depression stereotypes [49], perceived severity of depression [53], and maladaptive schemas or negative emotions [80].

Overall, the narrative synthesis showed that the included studies assessed a heterogeneous set of outcomes with different substantive meanings. Across most studies, parasocial engagement with social media influencers was positively associated with the outcomes assessed, although the substantive interpretation of these positive associations differed by outcome type. For positive outcomes, positive associations generally indicated higher well-being or more adaptive functioning; for negative outcomes, positive associations generally indicated higher levels of problematic social media use, distress, or maladaptive cognitions.

### Meta-Analytic Results

**Figure 2.**
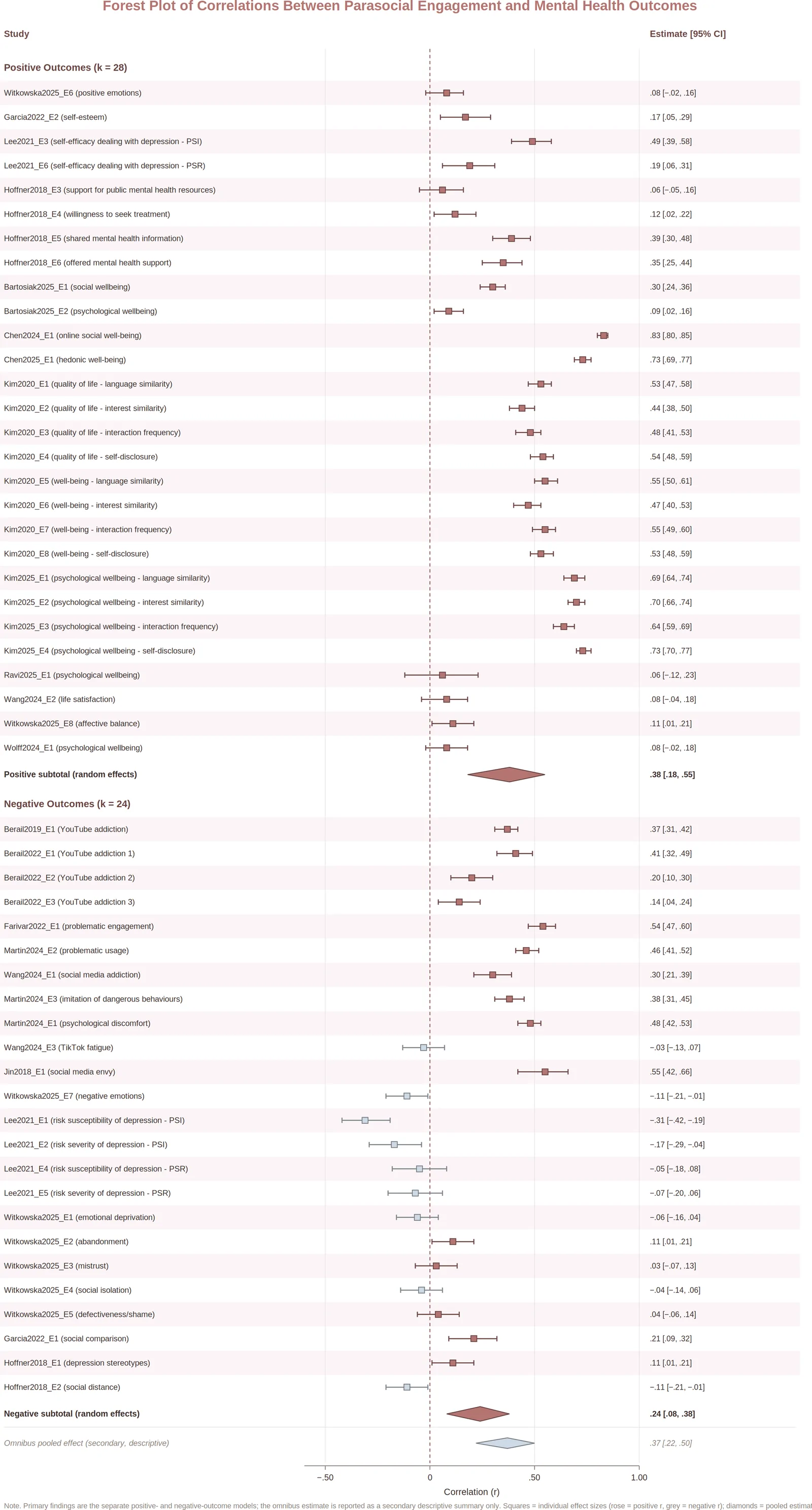
Forest plot of correlations between parasocial relationships and mental health outcomes across included studies [12,48-53,72-81].

Because multiple effect sizes were reported within some studies [12,48-50,74-77,79,80], a total of 52 effect sizes were extracted from 17 studies (Figure 2). Given the conceptual heterogeneity of the included mental health–related and psychosocial outcomes, the primary meta-analytic results were based on separate 3-level random-effects models for positive and negative outcomes.

As shown in Table 3, separate 3-level random-effects meta-analyses were conducted for positive and negative outcomes, given the conceptual heterogeneity of the included mental health–related and psychosocial outcomes. For positive outcomes, the 3-level random-effects meta-analysis (*k*=28; n=12) showed a significant association between influencer-directed parasocial engagement and positive outcomes (*β*=.406, SE=0.111, *z*=3.66; *P*<.001). The pooled correlation was *r*=0.38 (95% CI 0.18-0.55), indicating a moderate positive association.

**Table 3.** Summary of 3-level meta-analytic results for associations between parasocial relationships and mental health outcomes^a^.

Statistic	Positive outcomes	Negative outcomes
Main effect analysis		
*k*^b^ (effects)	28	24
Studies, n	12	10
Pooled *r*	0.38	0.24
95% CI	0.18-0.55	0.08-0.38
Heterogeneity statistics		
Cochran *Q*	1372.68	632.06
*df*	27	23
*P* value	<.001	<.001
τ² (level 3)	0.140	0.069
τ² (level 2)	0.009	0.012
*I*² (level 3)	0.94	0.85
*I*² (level 2)	0.06	0.15

a Level 3 variance reflects between-study heterogeneity, and level 2 variance reflects within-study heterogeneity.

b *k*: number of effect sizes.

Significant heterogeneity was observed for positive outcomes, *Q*(27)=1372.68; *P*<.001. Variance components indicated substantial between-study variance (τ²(3)=0.140) and smaller within-study variance across effect sizes (τ²(2)=0.009). The proportion of variance attributable to study-level differences was high (*I*²(3)=0.94), with additional heterogeneity at the effect-size level (*I*²(2)=0.06), indicating that associations varied primarily across studies.

For negative outcomes, the 3-level random-effects meta-analysis (*k*=24; n=10) also showed a significant association between influencer-directed parasocial engagement and negative outcomes (*β*=.245, SE=0.093; *z*=2.65; *P*=.008). The pooled correlation was *r*=0.24 (95% CI 0.08-0.38), indicating a small to moderate positive association.

Significant heterogeneity was also observed for negative outcomes, *Q*(23)=632.06; *P*<.001. Variance components indicated substantial between-study variance (*τ*²(3)=0.069) and additional within-study variance across effect sizes (*τ²*(2)=0.012). The proportion of variance attributable to study-level differences was high (*I*²(3)=0.85), with further heterogeneity at the effect-size level (*I*²(2)=0.15), indicating that associations varied both across studies and across outcomes within studies.

To evaluate whether a multilevel model was necessary, the 3-level model was compared with a conventional 2-level random-effects model separately for positive and negative outcomes. For positive outcomes, the 3-level model showed a better fit to the data (AIC=−3.85) than the 2-level model (AIC=37.66). For negative outcomes, the 3-level model also showed a better fit (AIC=−4.59) than the 2-level model (AIC=32.20). These results supported the use of a 3-level meta-analytic approach to account for dependency among effect sizes.

For descriptive purposes, we also estimated an omnibus 3-level random-effects model including all eligible effect sizes (*k*=52; n=17). This model was used to summarize the broader evidence base. However, because the included outcomes differed in valence and conceptual meaning, this overall estimate was not interpreted as a single beneficial or harmful mental health effect. Rather, it showed a positive average association between influencer-directed parasocial engagement and mental health–related and psychosocial outcomes, *β*=.388, SE=0.084, *z*=4.64, *P*<.001, corresponding to *r*=0.37 (95% CI 0.22-0.50). This estimate should be interpreted descriptively as the average statistical association across a heterogeneous set of outcome measures. Variance decomposition indicated that 83.32% of the total variance was attributable to differences between studies, whereas 16.68% reflected variability between effect sizes within studies, supporting the presence of substantial clustering of effect sizes.

### Moderator and Subgroup Analyses

Moderator analyses were conducted using 3-level meta-regression models to examine whether the association varied across study and sample characteristics. The examined moderators included mental health outcome types, parasocial construct types, social media platform type, region (Western vs Eastern), age group (adolescents vs young adults vs adults), and gender composition. Outcome types significantly moderated the association, *QM*(3)=22.83; *P*<.001. Parasocial types also showed a significant moderating effect, *QM*(2)=7.60, *P*=.022, with stronger associations observed for PSI compared with other forms. Region significantly moderated the effect, *QM*(1)=4.54, *P*=.033, indicating larger effects in Eastern samples than in Western samples. Age group was also a significant moderator, *QM*(3)=16.01, *P*=.001, suggesting that the association varied across age stages. However, gender composition, *QM*(1)=0.46, *P*=.50, and social media platform types, *QM*(4)=6.26, *P*=.18, were not significant moderators.

**Figure 3.**
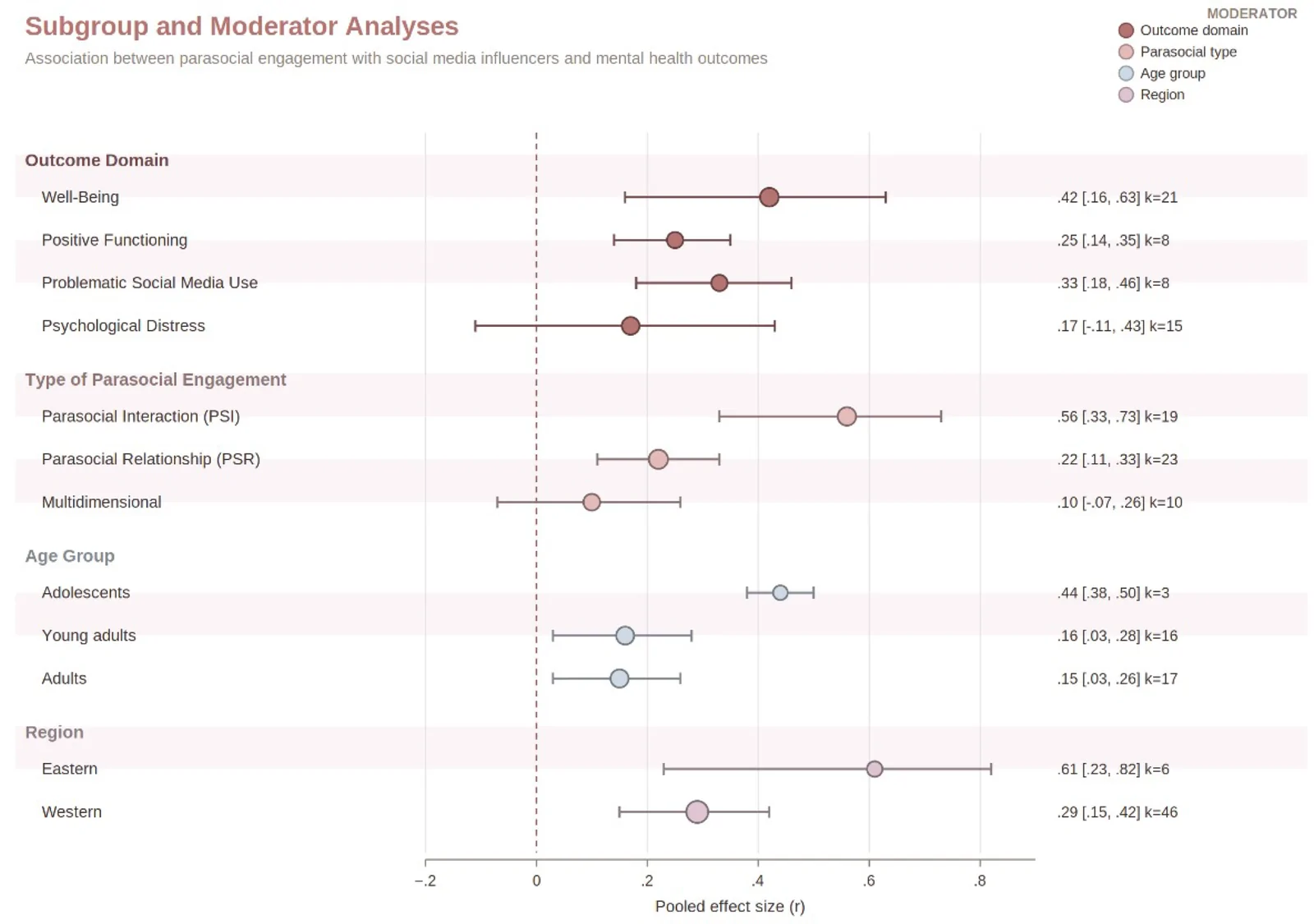
Subgroup meta-analysis of the association between parasocial relationships and mental health outcomes.

Subgroup analyses were conducted for significant moderators, including outcome type, parasocial type, age group, and region. The magnitude of the association varied across outcome types. The strongest effects were observed for well-being outcomes (*r*=0.42, 95% CI 0.16-0.63), followed by problematic social media use (*r*=0.33, 95% CI 0.18-0.46) and positive functioning (*r*=0.25, 95% CI 0.14-0.35), whereas effects for psychological distress were not significant. Differences were also found across parasocial constructs. The association was strongest for PSI (*r*=0.56, 95% CI 0.33-0.73), followed by PSR (*r*=0.22, 95% CI 0.11-0.33), while multidimensional measures showed a nonsignificant effect. Subgroup analyses by age group indicated that the association was strongest among adolescents (*r*=0.44, 95% CI 0.38-0.50) and smaller among young adults (*r*=0.16, 95% CI 0.03-0.28) and adults (*r*=0.15, 95% CI 0.03-0.26). Finally, the association differed by region, with larger effects observed in Eastern samples (*r*=0.61, 95% CI 0.23-0.82) than in Western samples (*r*=0.29, 95% CI 0.15-0.42; Figure 3 and Table 4).

**Table 4.** Subgroup analysis of outcome types, parasocial construct types, age groups, and regions.

Subgroup	*k*	*r*	95% CI	*P* value
Outcome types				
Problematic social media use	8	0.33	0.18 to 0.46	<.001
Psychological distress	15	0.17	−0.11 to 0.43	.23
Positive functioning	8	0.25	0.14 to 0.35	<.001
Well-being	21	0.42	0.16 to 0.63	.002
Parasocial constructs				
PSI^a^	19	0.56	0.33 to 0.73	<.001
PSR^b^	23	0.22	0.11 to 0.33	<.001
Multidimension	10	0.1	−0.07 to 0.26	.26
Age groups				
Adolescents	3	0.44	0.38 to 0.50	<.001
Young adults	16	0.16	0.03 to 0.28	.016
Adults	17	0.15	0.03 to 0.26	.017
Regions				
Eastern	6	0.61	0.23 to 0.82	.003
Western	46	0.29	0.15 to 0.42	<.001

a PSI: parasocial interaction.

b PSR: parasocial relationship.

### Publication Bias

**Figure 4.**
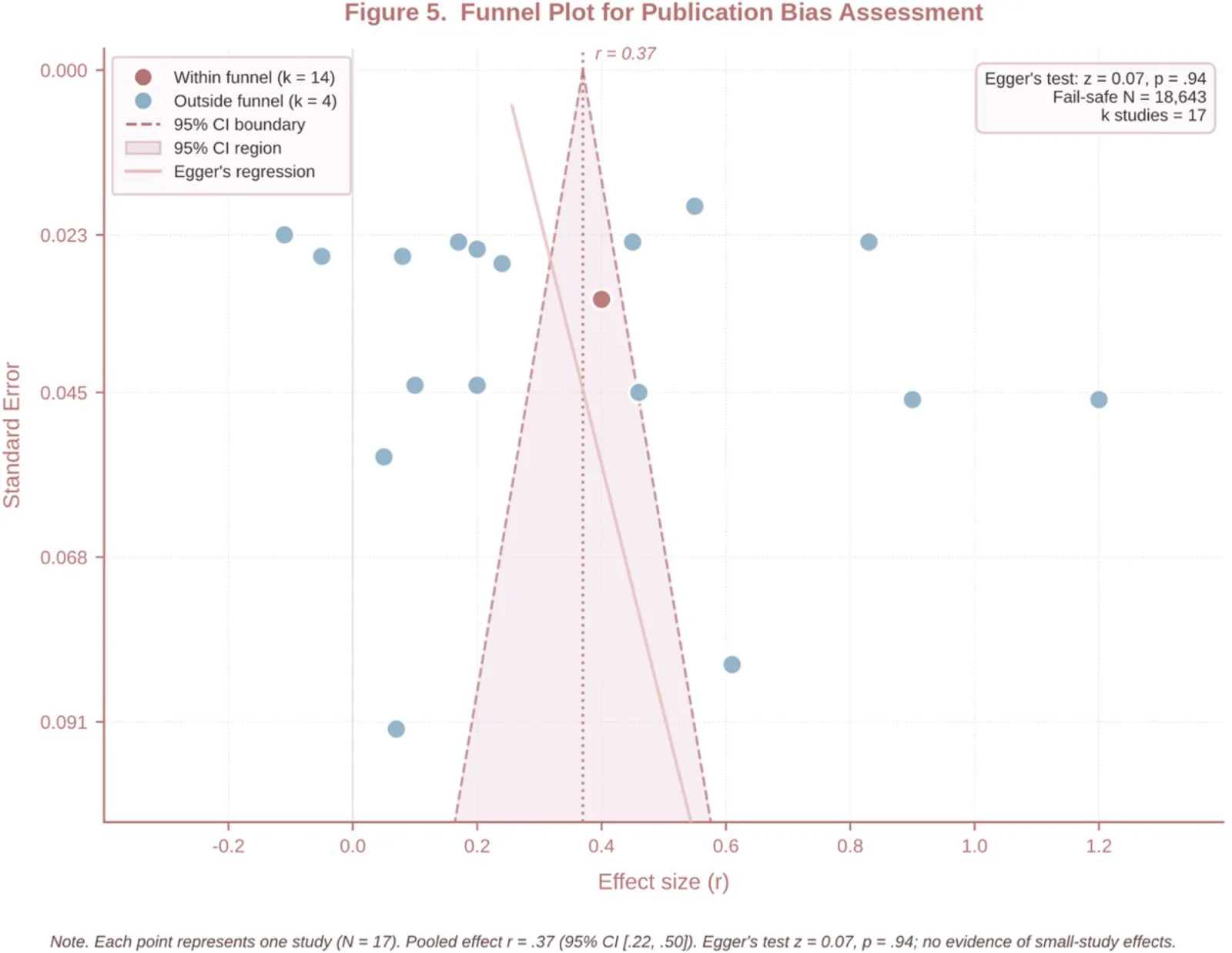
Funnel plot assessing publication bias in studies of parasocial relationships and mental health outcomes.

Publication bias was examined at the study level using funnel plots, Egger regression test, and fail-safe N. As shown in Figure 4, visual inspection of the funnel plot did not indicate substantial asymmetry. Egger regression test was not significant (*z*=0.07, *P*=.94), suggesting no evidence of small-study effects. Fail-safe N analysis using Rosenthal’s approach showed that 18,643 additional null studies would be required to reduce the overall effect to nonsignificance, indicating that the observed association was highly robust to potential publication bias.

### Sensitivity Analysis

Sensitivity analyses were conducted to examine the robustness of the findings. First, when the analysis was restricted to effect sizes directly reported as correlations (*k*=43), the pooled effect remained similar with the main analysis (*r*=0.40 and 0.37, respectively), indicating that the results were not driven by converted effect sizes. Second, leave-one-out analyses indicated that the pooled effect size remained stable across all iterations (range: *r*=0.33-0.39), suggesting that the overall result was not driven by any single study. Together, these findings suggest that the overall results were robust across different analytic decisions.

### Quality Assessment

**Figure 5.**
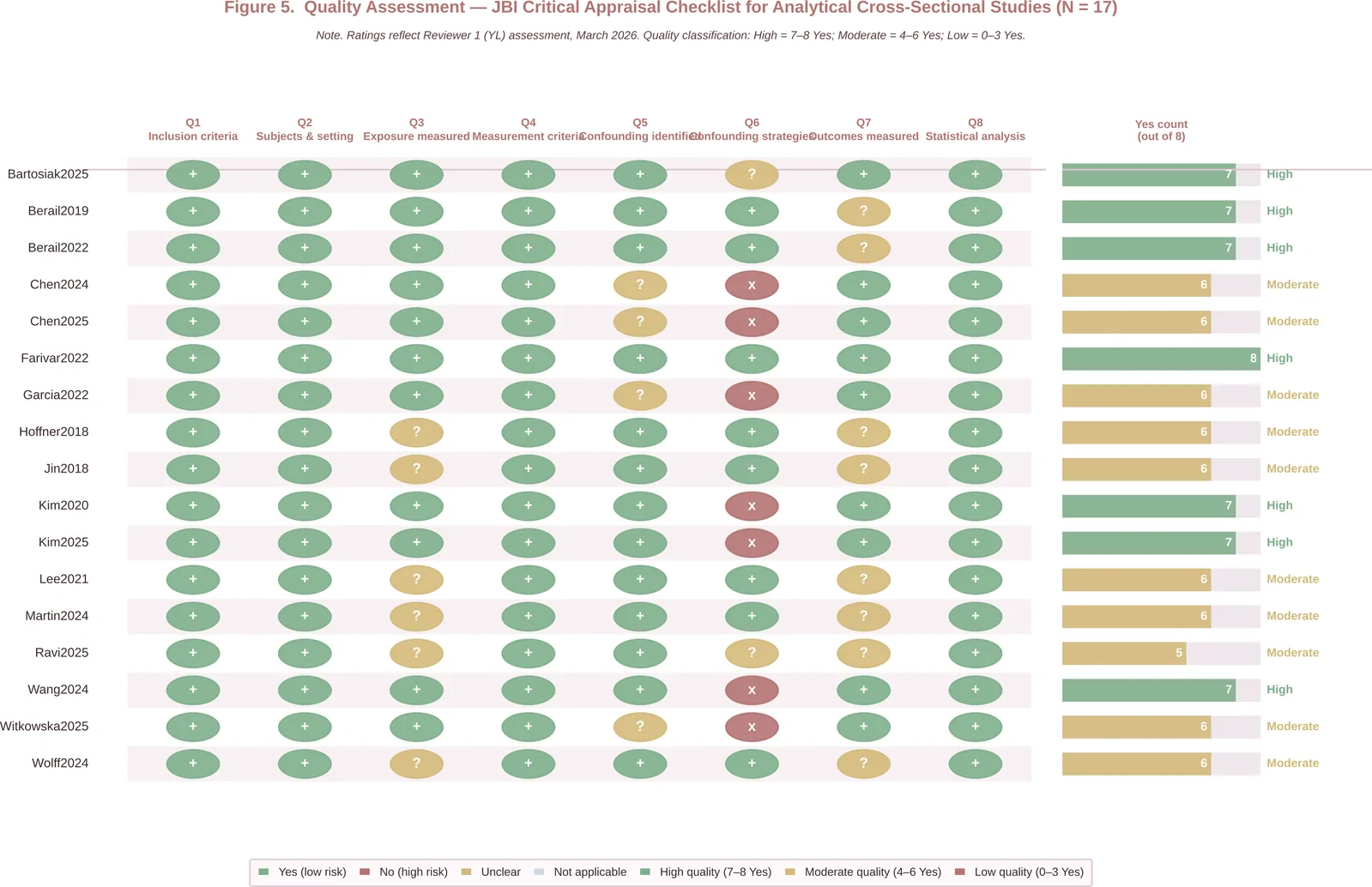
Quality appraisal of included studies across risk-of-bias criteria [12,48-53,72-81].

Methodological quality was assessed across all 17 included studies using the JBI Critical Appraisal Checklist for Analytical Cross-Sectional Studies, with each study rated across 8 criteria. Overall, the quality of the included studies was moderate to high. Seven studies were classified as high quality (scores of 7‐8 out of 8). The remaining 10 studies received moderate-quality ratings (scores of 4‐6). No studies were rated as low quality (Figure 5).

## Discussion

### Principal Findings

The present systematic review and 3-level meta-analysis is the first quantitative synthesis of the associations between parasocial engagement with social media influencers and mental health–related outcomes. Across 17 studies and 52 effect sizes, meta-analyses showed that parasocial engagement was moderately associated with positive outcomes (28 effect sizes) and showed a small to moderate association with negative outcomes (24 effect sizes). These positive associations have different substantive meanings depending on the outcome assessed: for positive outcomes, they indicate higher well-being or more adaptive functioning, whereas for negative outcomes, they indicate higher levels of problematic social media use or psychological distress. Furthermore, moderator analyses revealed that the strength and direction of the association were influenced by mental health outcome type, parasocial construct, age group, and cultural context. These findings suggest that rather than indicating a uniformly beneficial or harmful phenomenon, the findings suggest a conditional pattern in which the implications of influencer-directed parasocial bonds vary across outcome domains and participant contexts.

### Parasocial Engagement With Social Media Influencers as a Double-Edged Sword

The findings showed that parasocial engagement with social media influencers was associated with both positive and negative outcomes. In contemporary social media environments, influencers maintain a continuous and personalized presence through frequent self-disclosure, interactive communication, and algorithmically driven exposure, which may strengthen the perceived intimacy of these relationships [82]. As a result, parasocial engagement with influencers may function in ways similar to real social relationships, influencing emotional adjustment, behavior, and well-being [17]. Importantly, the findings indicate that parasocial engagement with influencers cannot be characterized as uniformly beneficial or harmful. Instead, the results support the view that influencer-directed parasocial bonds may function as a double-edged sword, being associated with both higher well-being and greater vulnerability.

One notable finding was that positive associations were generally larger and more consistent than negative associations. One possible explanation is that parasocial engagement with influencers often occurs in contexts that are voluntarily chosen and emotionally rewarding [75]. Individuals typically follow influencers they like, admire, or identify with, which may increase the likelihood of experiencing positive emotions such as inspiration, companionship, or enjoyment [17,83]. In addition, influencer content is often designed to be engaging, supportive, and aspirational, which may further promote positive affect and well-being [84]. Another possibility is that positive outcomes such as well-being and life satisfaction are more immediately responsive to perceived social connection, whereas negative outcomes such as psychological distress or problematic use may require additional vulnerability factors, such as social comparison or limited offline support [12]. This may explain why parasocial engagement is more consistently linked to positive functioning, while negative effects appear to depend more strongly on individual and contextual risk conditions.

According to the review, parasocial engagement was related to various positive mental health outcomes, such as higher levels of well-being [12], life satisfaction [81], and positive emotions [80]. The associations were generally stronger for well-being–related outcomes than for other forms of positive functioning. Other studies examined positive responses such as self-esteem [48], willingness to support public mental health resources [49], and self-efficacy in dealing with depression [53], but the effects for these outcomes tended to be smaller and less consistent. One possible explanation is that well-being–related outcomes reflect broader affective states that may be more immediately influenced by perceived social connection and emotional support from influencers. In social media environments, influencers are often perceived as relatable, authentic, and emotionally accessible [85], and perceived interaction and responsiveness may enhance followers’ sense of being valued and understood, thereby strengthening emotional attachment and perceived authenticity [86]. Forming a connection that resembles friendship with influencers may therefore foster feelings of companionship and belonging, which are closely linked to subjective well-being and life satisfaction [12,87]. In contrast, more specific outcomes such as self-esteem, self-efficacy, or behavioral intentions may depend on additional personal and contextual factors, which could explain the weaker associations observed for these outcomes.

Several theoretical perspectives may explain why parasocial engagement with influencers is associated with positive psychological outcomes. According to self-expansion theory, individuals are motivated to enhance and broaden their sense of self by incorporating others into their self-concept [88]. Influencers often present new lifestyles, values, and experiences, which may provide followers with opportunities for self-expansion through identification and imagined interaction [89]. Such self-expanding experiences have been associated with increased positive affect and well-being [90] and may therefore explain why stronger parasocial engagement is linked to higher well-being in social media contexts. In addition, self-determination theory suggests that parasocial engagement may satisfy basic psychological needs, particularly the need for relatedness [43]. When influencers are perceived as supportive and authentic, PSIs may provide a sense of connection that encourages well-being and adaptive behaviors such as help-seeking [44]. For individuals in identity-forming stages, influencers who model inclusive or affirming identities may also promote self-acceptance and belonging [45]. The presence of both beneficial and adverse pathways underscores the need to examine parasocial effects across diverse mental health outcomes rather than assuming a single direction of influence [46].

Despite these positive outcomes, parasocial engagement was also significantly associated with negative mental health outcomes, including social media addiction [50], emotional distress, and maladaptive cognitions [80]. The effects for social media addiction were larger than for other negative outcomes, which may suggest that parasocial bonds are more directly linked to patterns of repeated engagement with influencer content [73,91]. Strong parasocial engagement may be linked to frequent checking, continued exposure, and emotional investment in influencers, which are closely related to excessive or compulsive social media use. In contrast, outcomes such as distress or maladaptive beliefs may depend on additional individual vulnerabilities or contextual factors [92], which could explain the relatively smaller effects observed for these outcomes.

Several theoretical perspectives may explain why parasocial engagement with influencers is linked to negative outcomes. Social comparison theory suggests that repeated exposure to idealized self-presentations may increase upward comparison, leading to dissatisfaction, reduced self-worth, and negative affect [27]. Because parasocial engagement involves perceived intimacy, comparisons with influencers may be experienced as personally relevant, which may intensify emotional responses. Self-discrepancy theory further proposes that exposure to idealized standards may increase the gap between actual and ideal selves, contributing to anxiety, depressive symptoms, or feelings of inadequacy [31,93]. In addition, strong parasocial involvement may foster emotional dependency or replace offline social interaction, which may increase loneliness or problematic media use over time [54]. These processes may be particularly pronounced in social media environments, where influencers are highly visible and continuously present, increasing the likelihood of repeated comparison and emotional investment.

### Differences Across Parasocial Constructs and Demographic Contexts

Although the present review uses the term parasocial engagement as an umbrella label for conceptual consistency, it is important to acknowledge that the included studies assessed different parasocial constructs, including PSI, PSR, and multidimensional measures that combined multiple components. The type of parasocial construct significantly moderated the association, with the strongest effects observed for PSI, followed by PSR, whereas multidimensional parasocial measures showed weaker effects. On the one hand, this finding supports theoretical distinctions between PSI and PSR, which propose that PSI occurs during media exposure, whereas PSR reflects a more enduring relational bond that extends beyond specific media encounters [54,55]. As PSI captures momentary feelings of engagement and perceived reciprocity, it may be more closely linked to short-term emotional and behavioral responses. Many mental health–related outcomes, such as mood, well-being, or problematic media use, are sensitive to situational emotional experiences [94]; therefore, measures assessing momentary interaction may show stronger associations than broader relationship-level constructs.

Differences were also observed across age groups, with the strongest effects observed among adolescents and weaker effects among young adults and adults. Adolescence represents a developmental period characterized by heightened sensitivity to social evaluation, identity formation, and peer comparison [95]. During this stage, young people may be particularly responsive to social media influences, as suggested by previous research showing that adolescents are more susceptible to online social comparison, feedback, and peer-related experiences [5,96,97]. Because influencer content often provides models of appearance, lifestyle, and success, parasocial involvement may intensify these developmental processes, especially when offline social support is limited. Taken together, these findings suggest that adolescence may be a period in which parasocial engagement has both substantial potential benefits and increased psychological risks.

Regional differences were also observed, with larger effects in Eastern samples than in Western samples. One possible explanation is that parasocial engagement may play a different role in collectivistic cultural contexts [19], where social harmony, belonging, and identification with admired figures are more strongly emphasized [98]. Individuals from interdependent cultural contexts are more likely to attend to social and situational information when interpreting others’ behavior [99], which may make media figures more psychologically salient. In such contexts, influencers may be perceived as more meaningful social reference figures, and parasocial bonds may therefore carry stronger emotional consequences [100]. Taken together, these findings suggest that cultural context should be considered an important boundary condition when evaluating the psychological effects of parasocial engagement.

### Strengths and Limitations

The present meta-analysis has several strengths. First, by applying a 3-level meta-analytic model, the analysis accounted for the dependency of multiple effect sizes within studies, allowing for more accurate estimation of the overall effect [64]. In addition, the inclusion of moderator and subgroup analyses made it possible to identify important boundary conditions, including outcome type, parasocial construct, age group, and cultural context.

Several limitations should also be noted. First, the number of available studies was relatively small, and the included studies varied considerably in outcome measures and operationalizations of parasocial engagement, contributing to substantial heterogeneity. The included outcomes also differed in conceptual meaning, ranging from well-being and positive functioning to problematic social media use and psychological distress. Therefore, these outcomes should not be interpreted as interchangeable indicators of a single overarching mental health construct. Although the omnibus model provides a useful descriptive summary of the broader evidence base, it should not be interpreted as showing that parasocial engagement is generally beneficial or harmful for mental health. This limitation also affects the interpretation of subgroup and moderator analyses. Although well-being and problematic social media use were examined in a sufficient number of studies to allow more stable estimates, most other outcome categories were represented by only a small number of studies and showed substantial variability across measures. As a result, domain-specific findings, particularly those for Positive Functioning and Psychological Distress, should be interpreted cautiously. Future research would benefit from more consistent measurement of mental health–related outcomes and from clearer theoretical distinctions between outcome domains, which would allow more precise tests of when parasocial engagement is associated with adaptive versus maladaptive correlates.

Second, the included studies varied in how parasocial engagement was operationalized, including PSR, PSI, and multidimensional parasocial measures. Although the present review used parasocial engagement as an umbrella term to synthesize this broader evidence base, these constructs are conceptually distinguishable and should not be interpreted as interchangeable. This construct heterogeneity may have contributed to variation in effect sizes and limits the precision with which the present findings can be attributed to any single parasocial construct. Future research should distinguish more clearly between different dimensions of influencer-directed parasocial engagement, including perceived interaction, emotional attachment, identification, and dependency.

Third, most included studies used cross-sectional designs, which limits potential causal and directional interpretation. The observed associations should therefore not be interpreted as evidence that parasocial engagement causes changes in mental health–related outcomes. It is also possible that individuals with particular psychological characteristics, such as lower well-being, loneliness, or psychological distress, are more likely to develop stronger parasocial engagement [101]. Future longitudinal and experimental studies are needed to clarify the temporal direction, potential bidirectionality, and mechanisms underlying these associations.

Finally, most samples consisted of adolescents or young adults and were conducted primarily in Western countries, which may limit the generalizability of the findings. Future research with more diverse samples and more consistent measurement of parasocial constructs and psychological outcomes is needed to clarify the links between parasocial engagement and mental health.

### Implications and Future Directions

This review has important implications for digital mental health and media literacy. The results suggest that parasocial engagement can support well-being in some cases but may also contribute to problematic engagement or emotional distress when involvement becomes excessive or comparison-based. These findings highlight the importance of promoting balanced and reflective engagement with influencer content. Educational and prevention programs may benefit from helping young people understand how parasocial processes operate and how social comparison and emotional dependency may arise in digital environments. At the same time, influencers themselves may play a role in shaping healthier online norms by modeling authenticity, diversity, and realistic self-presentation.

Beyond implications, the review points to several directions for future research. First, different dimensions of parasocial involvement (eg, PSI vs PSR) may have different psychological consequences. Future work should distinguish between perceived interaction, emotional attachment, identification, and dependency, rather than treating parasocial engagement as a unitary construct. Such distinctions may help explain inconsistencies in previous findings and improve theoretical precision.

Second, given the increasing prominence of influencers in social media in everyday life, particularly among adolescents and young adults [77], understanding the psychological consequences of parasocial engagement is essential. Nevertheless, the number of studies focusing specifically on adolescents was relatively limited, although adolescence represents a critical developmental period of sensitivity to social media during adolescence [97]. Future research should examine age-related differences using longitudinal designs and consider how developmental needs, peer dynamics, and identity processes shape the formation and impact of parasocial bonds with influencers.

In addition, more longitudinal studies are needed to clarify the dynamic nature of parasocial processes. Most included studies used cross-sectional designs and relied on self-report measures, limiting conclusions about causality. It remains unclear whether strong parasocial engagement leads to changes in mental health, or whether individuals with certain psychological characteristics are more likely to form intense parasocial bonds. Longitudinal and experimental research is needed to clarify the direction of these associations. Besides, parasocial engagement develops gradually through repeated exposure and perceived interaction, and momentary experiences of interaction may be particularly influential for emotional responses [55]. Longitudinal designs would help to capture how short-term interaction experiences accumulate into more stable parasocial engagement and how these processes relate to mental health outcomes over time.

Finally, although the overall methodological quality of the included studies was generally good, several limitations should be noted. In some studies, the identification and control of potential confounding variables were not clearly reported, and information regarding the validity or reliability of outcome measures was occasionally limited. Future studies should use more rigorous designs, including clearer reporting of confounders, and the use of well-validated measures, to strengthen the evidence base.

### Conclusions

This meta-analysis provides the first quantitative synthesis of the association between parasocial engagement with social media influencers and mental health–related outcomes. The central finding is that parasocial engagement should not be understood as uniformly beneficial or harmful. Rather, its psychological meaning depends on what outcome is being considered. Parasocial engagement was associated with both positive and negative outcomes, but positive outcomes, such as well-being, were generally stronger and more consistent. The findings further suggest that the psychological impact of parasocial engagement varies depending on the type of parasocial construct, developmental stage, and cultural context, with stronger effects observed for PSI, adolescent samples, and Eastern samples. Future research should examine a broader range of mental health outcomes and focus on longitudinal and mechanism-based designs to clarify when and for whom parasocial engagement is most beneficial or harmful.

## Supplementary material

10.2196/96331 Multimedia Appendix 1 Sample search strings.

10.2196/96331 Multimedia Appendix 2 Overview of study characteristics in the included studies (N=17).

10.2196/96331 Checklist 1 PRISMA checklist.

## Abbreviations

AIC: Akaike Information Criterion
JBI: Joanna Briggs Institute
PRISMA: Preferred Reporting Items for Systematic Reviews and Meta-Analyses
PSI: parasocial interaction
PSR: parasocial relationship
