# Supplementary material for: Parasocial Engagement With Social Media Influencers and Mental Health Outcomes: Systematic Review and Meta-Analysis
Source: JMIR Ment Health. 2026 Aug 20;13:e96331. doi: 10.2196/96331 (PMC13492728; doi:10.2196/96331)
Supplement: Supplementary file 1 [file mental-v13-e96331-s001.docx]

**Multimedia Appendix 1**

**Database Search Strategy**

The following combination of search terms was used across databases, with adaptations to database-specific syntax and controlled vocabulary:

(parasocial relationship OR parasocial interaction OR parasocial bond OR parasocial attachment OR one-sided relationship OR audience-creator relationship)

AND

(social media influencer OR social media celebrity OR influencer OR streamer OR YouTuber OR Instagram OR TikTok OR social media figure OR online influencer OR virtual influencer)

AND

(depress* OR lonel* OR social isolate* OR mental* OR well-being OR psychological health OR anxie* OR panic* OR self-esteem OR ocd OR obsessive-compulsive* OR bipolar* OR ptsd OR post-traumatic* OR stress OR dissociat* OR phobi* OR self injur* OR suicid*)

***Notes.*** * were used to capture variations of keywords (e.g., depress* = depression, depressive); Search strings were adapted for each database (e.g., Web of Science, PsycINFO) to account for differences in syntax and indexing; No restrictions were applied to publication year and language.
