# Supplementary material for: Parasocial Engagement With Social Media Influencers and Mental Health Outcomes: Systematic Review and Meta-Analysis
Source: JMIR Ment Health. 2026 Aug 20;13:e96331. doi: 10.2196/96331 (PMC13492728; doi:10.2196/96331)
Supplement: Supplementary file 2 [file mental-v13-e96331-s003.docx]

**PRISMA 2020 Checklist**

| **Section and Topic** | **Item #** | **Checklist item** | **Location where item is reported** |
| --- | --- | --- | --- |
| **TITLE** | | |  |
| Title | 1 | Identify the report as a systematic review. | Title, p. 1 |
| **ABSTRACT** | | |  |
| Abstract | 2 | See the PRISMA 2020 for Abstracts checklist. | Not included in this manuscript version (abstract not provided in the reviewed document) |
| **INTRODUCTION** | | |  |
| Rationale | 3 | Describe the rationale for the review in the context of existing knowledge. | Introduction, paragraphs 1–8 (pp. 1–4): Reviews theoretical frameworks (PSR/PSI, social comparison theory, self-discrepancy theory, SDT), identifies empirical heterogeneity and gaps in existing literature. |
| Objectives | 4 | Provide an explicit statement of the objective(s) or question(s) the review addresses. | The Present Study section (p. 4): Three research objectives (RO1–RO3) explicitly stated — pooled effect size estimation, moderator analyses, and evidence quality evaluation. |
| **METHODS** | | |  |
| Eligibility criteria | 5 | Specify the inclusion and exclusion criteria for the review and how studies were grouped for the syntheses. | Method – Eligibility Criteria section and Table 1: PECO framework with Population, Exposure, Comparator, Outcomes, Study design, Language, and Exclusion criteria fully specified. |
| Information sources | 6 | Specify all databases, registers, websites, organisations, reference lists and other sources searched or consulted to identify studies. Specify the date when each source was last searched or consulted. | Method – Information Sources and Search Strategy section (p. 5): Seven databases listed (PsycINFO, EMBASE, MEDLINE, ERIC, PubMed, Web of Science, Scopus), plus Google Scholar (first 200 results), reference list hand-searching, and author contact. Searches from inception to final search date. |
| Search strategy | 7 | Present the full search strategies for all databases, registers and websites, including any filters and limits used. | Method – Information Sources and Search Strategy: Strategy structured around three conceptual domains (parasocial construct, social media influencers, mental health outcomes). Full database-specific search strings reported in Supplemental Material II. |
| Selection process | 8 | Specify the methods used to decide whether a study met the inclusion criteria of the review, including how many reviewers screened each record and each report retrieved, whether they worked independently, and if applicable, details of automation tools used in the process. | Method – Study Selection (p. 5): Two reviewers (FQL and YXL) independently screened titles/abstracts and full texts using Rayyan software. Standardised screening form based on PECO criteria. Disagreements resolved by structured discussion. Inter-rater reliability quantified using Cohen’s κ (≥ .80 threshold). |
| Data collection process | 9 | Specify the methods used to collect data from reports, including how many reviewers collected data from each report, whether they worked independently, any processes for obtaining or confirming data from study investigators, and if applicable, details of automation tools used in the process. | Method – Data Extraction (p. 6): Standardised extraction form piloted on five studies. Discrepancies resolved by discussion or third-party adjudication. Missing data sought from corresponding authors via up to two email requests over four weeks. |
| Data items | 10a | List and define all outcomes for which data were sought. Specify whether all results that were compatible with each outcome domain in each study were sought (e.g. for all measures, time points, analyses), and if not, the methods used to decide which results to collect. | Method – Data Extraction (p. 6): Outcome characteristics specified (mental health outcome type, measure, item count, reliability). Multiple effect sizes from same study treated as dependent. Results section details positive outcomes (wellbeing, life satisfaction, self-esteem, etc.) and negative outcomes (addiction, distress, envy, maladaptive schemas, etc.). |
|  | 10b | List and define all other variables for which data were sought (e.g. participant and intervention characteristics, funding sources). Describe any assumptions made about any missing or unclear information. | Method – Data Extraction (p. 6): Five categories extracted: (a) study characteristics, (b) sample characteristics, (c) exposure characteristics (PSR/PSI construct, measure, platform, influencer type), (d) outcome characteristics, (e) statistical information (correlations, regression coefficients, means, SDs). |
| Study risk of bias assessment | 11 | Specify the methods used to assess risk of bias in the included studies, including details of the tool(s) used, how many reviewers assessed each study and whether they worked independently, and if applicable, details of automation tools used in the process. | Method – Risk of Bias Assessment (p. 6): JBI Critical Appraisal Checklist for Analytical Cross-Sectional Studies applied. Two reviewers assessed independently; disagreements resolved through discussion. Overall quality score calculated as proportion of criteria met. |
| Effect measures | 12 | Specify for each outcome the effect measure(s) (e.g. risk ratio, mean difference) used in the synthesis or presentation of results. | Method – Effect Size Metric (p. 6): Pearson’s r adopted as common metric. Other metrics (β, Cohen’s d, odds ratios) converted using established formulae. Fisher’s z transformation applied; back-transformed for reporting. Benchmarks: r = .10 (small), .30 (medium), .50 (large). |
| Synthesis methods | 13a | Describe the processes used to decide which studies were eligible for each synthesis (e.g. tabulating the study intervention characteristics and comparing against the planned groups for each synthesis (item #5)). | Method – Narrative Synthesis (p. 8): Outcome categories with fewer than 3 studies or excessive heterogeneity underwent narrative synthesis following SWiM guidelines. All other eligible studies included in meta-analysis. |
|  | 13b | Describe any methods required to prepare the data for presentation or synthesis, such as handling of missing summary statistics, or data conversions. | Method – Effect Size Metric (p. 6): Effect sizes converted to r using established formulae [55]. Fisher’s z transformation applied for analysis. Missing data sought from authors via email. |
|  | 13c | Describe any methods used to tabulate or visually display results of individual studies and syntheses. | Results: Table 2 (study characteristics), Table 3 (meta-analytic results), Table 4 (subgroup analyses), Figure 1 (PRISMA flow diagram), Figure 2 (study characteristics overview), Figure 3 (forest plot), Figure 4 (subgroup forest plots), Figure 5 (funnel plot), Figure 6 (quality appraisal). |
|  | 13d | Describe any methods used to synthesize results and provide a rationale for the choice(s). If meta-analysis was performed, describe the model(s), method(s) to identify the presence and extent of statistical heterogeneity, and software package(s) used. | Method – Meta-analytic Model (pp. 6–7): Three-level random-effects model (effect sizes nested within studies). REML estimator. R (v4.5.1) with metafor package. Model fit evaluated via AIC and likelihood ratio tests comparing 2-level vs. 3-level models. |
|  | 13e | Describe any methods used to explore possible causes of heterogeneity among study results (e.g. subgroup analysis, meta-regression). | Method – Moderator Analyses (p. 7): Mixed-effects meta-regression within three-level framework. Six pre-specified moderators: outcome domain, parasocial construct type, age group, gender composition (continuous), social media platform, cultural region. Bonferroni-corrected threshold p < .006. Minimum k = 3 per subgroup. |
|  | 13f | Describe any sensitivity analyses conducted to assess robustness of the synthesized results. | Method – Sensitivity Analyses (p. 8): Leave-one-out analyses; restriction to high-quality studies (≥ 70% JBI); restriction to validated PSR/PSI measures; restriction to peer-reviewed articles; re-analysis using only reported (unconverted) correlations; effect size source tested as categorical moderator. |
| Reporting bias assessment | 14 | Describe any methods used to assess risk of bias due to missing results in a synthesis (arising from reporting biases). | Method – Assessment of Publication Bias (pp. 7–8): Funnel plot visual inspection, Egger’s regression test (p < .10), trim-and-fill procedure, limit meta-analysis method, and selection model approaches. Applied when k ≥ 10. |
| Certainty assessment | 15 | Describe any methods used to assess certainty (or confidence) in the body of evidence for an outcome. | Not explicitly reported. No formal certainty-of-evidence assessment (e.g., GRADE) was described. |
| **RESULTS** | | |  |
| Study selection | 16a | Describe the results of the search and selection process, from the number of records identified in the search to the number of studies included in the review, ideally using a flow diagram. | Results (p. 9) and Figure 1: 1,450 records imported; 41 duplicates removed; 1,409 screened; 1,364 excluded at title/abstract; 45 full-text assessed; 28 excluded; 17 studies included. PRISMA flow diagram provided. |
|  | 16b | Cite studies that might appear to meet the inclusion criteria, but which were excluded, and explain why they were excluded. | Results (p. 9): 28 full-text articles excluded (mentioned but individual exclusion reasons not detailed in the main text; referenced via Figure 1). |
| Study characteristics | 17 | Cite each included study and present its characteristics. | Results – Study Characteristics (pp. 9–10) and Table 2: All 17 studies cited with details on design, location, sample characteristics, parasocial construct/measure, influencer/platform type, outcomes, and analytic methods. |
| Risk of bias in studies | 18 | Present assessments of risk of bias for each included study. | Results – Quality Assessment (p. 12) and Figure 6: JBI checklist results presented. Six studies rated high quality (7–8/8), ten moderate (4–6), none low quality. Figure 6 shows criteria-level ratings for each study. |
| Results of individual studies | 19 | For all outcomes, present, for each study: (a) summary statistics for each group (where appropriate) and (b) an effect estimate and its precision (e.g. confidence/credible interval), ideally using structured tables or plots. | Results and Figure 3: Forest plot displays individual effect sizes with CIs for all 52 effects from 17 studies. Table 2 provides study-level detail. |
| Results of syntheses | 20a | For each synthesis, briefly summarise the characteristics and risk of bias among contributing studies. | Results (pp. 9–12): Study characteristics summarised for overall, positive, and negative outcome syntheses. Quality assessment described in Quality Assessment section. |
|  | 20b | Present results of all statistical syntheses conducted. If meta-analysis was done, present for each the summary estimate and its precision (e.g. confidence/credible interval) and measures of statistical heterogeneity. If comparing groups, describe the direction of the effect. | Results – Meta-Analytic Results (pp. 10–11) and Table 3: Overall r = .37 [.22, .50]; positive outcomes r = .38 [.18, .55]; negative outcomes r = .24 [.08, .38]. Q, τ², I² at Levels 2 and 3 reported. AIC and likelihood ratio test for model comparison. |
|  | 20c | Present results of all investigations of possible causes of heterogeneity among study results. | Results – Moderator and Subgroup Analyses (p. 11), Table 4, and Figure 4: Significant moderators: outcome type (QM = 22.83, p < .001), parasocial construct (QM = 7.60, p = .022), region (QM = 4.54, p = .033), age group (QM = 16.01, p = .001). Non-significant: gender, platform. |
|  | 20d | Present results of all sensitivity analyses conducted to assess the robustness of the synthesized results. | Results – Sensitivity Analysis (p. 12): Restricting to reported correlations (k = 43): r = .40 [.23, .54]. Leave-one-out range: r = .33–.39. Results stable across analytic decisions. |
| Reporting biases | 21 | Present assessments of risk of bias due to missing results (arising from reporting biases) for each synthesis assessed. | Results – Publication Bias (p. 12) and Figure 5: Funnel plot shows no substantial asymmetry. Egger’s test non-significant (z = 0.07, p = .94). Rosenthal’s fail-safe N = 18,643. |
| Certainty of evidence | 22 | Present assessments of certainty (or confidence) in the body of evidence for each outcome assessed. | Not reported. No formal certainty-of-evidence assessment (e.g., GRADE) was presented. |
| **DISCUSSION** | | |  |
| Discussion | 23a | Provide a general interpretation of the results in the context of other evidence. | Discussion (pp. 13–16): Results interpreted in relation to social comparison theory, self-discrepancy theory, self-expansion theory, SDT, and prior meta-analyses on social media and mental health. |
|  | 23b | Discuss any limitations of the evidence included in the review. | Discussion – Strengths and Limitations (p. 17): Small number of studies, heterogeneous outcome measures, predominantly cross-sectional designs, self-report measures, limited samples (mainly young adults, Western countries). |
|  | 23c | Discuss any limitations of the review processes used. | Discussion – Strengths and Limitations (p. 17): Limited study pool noted. Variability in PSR operationalisations. Cross-sectional designs preclude causal inference. No GRADE assessment. |
|  | 23d | Discuss implications of the results for practice, policy, and future research. | Discussion – Implications and Future Directions (pp. 16–17): Recommendations for media literacy programmes, distinguishing PSI/PSR dimensions, longitudinal designs, adolescent-focused research, and more rigorous measurement. |
| **OTHER INFORMATION** | | |  |
| Registration and protocol | 24a | Provide registration information for the review, including register name and registration number, or state that the review was not registered. | Method – Protocol and Registration (p. 5): Prospectively registered on PROSPERO (Registration number: CRD420261291637). |
|  | 24b | Indicate where the review protocol can be accessed, or state that a protocol was not prepared. | Method – Protocol and Registration (p. 5): Protocol registered on PROSPERO (accessible via PROSPERO database). Supplemental Material I referenced for PRISMA checklist. |
|  | 24c | Describe and explain any amendments to information provided at registration or in the protocol. | Not explicitly reported in the manuscript. |
| Support | 25 | Describe sources of financial or non-financial support for the review, and the role of the funders or sponsors in the review. | Not reported in the reviewed manuscript. |
| Competing interests | 26 | Declare any competing interests of review authors. | Not reported in the reviewed manuscript. |
| Availability of data, code and other materials | 27 | Report which of the following are publicly available and where they can be found: template data collection forms; data extracted from included studies; data used for all analyses; analytic code; any other materials used in the review. | Not explicitly reported in the reviewed manuscript. |

*From: Page MJ, McKenzie JE, Bossuyt PM, Boutron I, Hoffmann TC, Mulrow CD, et al. The PRISMA 2020 statement: an updated guideline for reporting systematic reviews. BMJ 2021;372:n71. doi: 10.1136/bmj.n71. This work is licensed under CC BY 4.0.*
